# Effect of Ni Element in Self-Shielded Flux-Cored Wires on the Microstructural and Mechanical Property Evolutions of X80 Pipeline Steel Girth Welds

**DOI:** 10.3390/ma19102162

**Published:** 2026-05-21

**Authors:** Shujun Jia, Chengwu Cui, Chunliang Mao, Gang Liu, Qingyou Liu

**Affiliations:** 1 Engineering Structural Steel Research Institute, Central Iron and Steel Research Institute Company Limited, Beijing 100081, China; mcliang163@163.com (C.M.); westbrant@163.com (G.L.);; 2 China Petroleum Pipeline Research Institute Company Limited, Langfang 065000, China; swpuchengwucui@163.com

**Keywords:** self-shielded flux-cored wire, Ni content, microstructure, N element, mechanical property

## Abstract

In the present work, eleven self-shielded flux-cored wires with nickel (Ni) contents ranging from 1.42 wt.% to 4.02 wt.% were designed for the semi-automatic welding of X80 pipeline steel. The effects of Ni on the microstructural evolution and mechanical properties of the weld metal were investigated. The results indicate that when the Ni content is below 2.06 wt.%, the microstructures of both the solidification zone and the inter-pass reheating zone are dominated by coarse granular bainite and martensite/austenite (M/A) constituents. As the Ni content increases from 2.06 wt.% to 3.73 wt.%, the microstructure transforms to fine lath bainite with M/A constituents characterized by low content, small size, and uniform distribution. When the Ni content reaches 3.73 wt.%, the microstructure becomes almost fully bainite. Furthermore, with increasing the Ni content, both the yield strength and tensile strength of the weld metal increase from ~600 MPa to ~700 MPa and from ~660 MPa to ~730 MPa, respectively. However, the impact energy at −20 °C of the weld metal initially increases and then decreases, reaching a peak of ~110 J with the lowest degree of dispersion at a Ni content of approximately 3.73 wt.%. When the Ni content exceeds 3.73 wt.%, the ductility decreases slightly. Further analyses indicate that the synergistic effects of Ni in refining the microstructure and reducing the activity coefficient and solubility of nitrogen (N) jointly contribute to the impact toughness of the weld metal.

## 1. Introduction

Owing to its cost-effectiveness and high transport capacity, pipeline transport has become the primary method for global oil and gas transmission [1,2,3,4,5]. The growing societal demand for energy is driving pipeline construction toward longer distances, higher pressures, larger diameters, and higher steel grades [6,7,8,9,10]. Welded joints of long-distance oil and gas pipelines are inherently heterogeneous and prone to welding defects, which are the primary causes of pipeline failure [5,11,12,13,14,15,16,17]. Therefore, high-quality welding technologies are critical to ensuring that welded joints match the superior mechanical properties of high-strength pipeline steels.

Automatic welding is the mainstream method for long-distance pipeline construction, supplemented by semi-automatic processes (gas-shielded or self-shielded flux-cored welding) and manual arc welding [12,18,19,20]. Self-shielded flux-cored wire offers unique advantages, including no external shielding gas requirement, all-position operability, and strong wind resistance [18]. For pipeline welding in complex field environments, such as mountainous and desert regions, the hybrid process combining semi-automatic self-shielded flux-cored welding and manual arc welding is widely adopted due to its high efficiency and operational flexibility [18]. However, there are still some problems with self-shielded flux-cored wire welding, such as the easy generation of spatter during the welding process due to the gas-forming agent in the core of the wire, unstable impact toughness of the weld, and a large fluctuation range [21]. During welding, the droplet transition mode directly affects the degree of spatter, which could be reduced by adjusting the welding heat input [22].

The metallurgical environment of self-shielded welding is far more severe than that of gas metal arc welding. The “semi-open” molten pool allows atmospheric N_2_ to dissolve as atomic nitrogen, resulting in weld nitrogen contents as high as 200–500 ppm. During cooling, excess free nitrogen forms N_2_ pores that degrade strength, toughness, and fatigue performance. Over time, supersaturated free nitrogen precipitates as brittle Fe_4_N, further increasing weld brittleness. To address these issues, Al is typically introduced into self-shielded flux-cored wire for the purpose of decreasing nitrogen solubility in the weld metal [23]. However, when the Al content increases, more and larger AlN particles would appear, which could serve as the crack initiation, reducing the weld toughness [23]. As a ferrite-stabilizing element, Al promotes the formation of coarse proeutectoid ferrite. This microstructure, in turn, increases the weld’s susceptibility to cleavage fracture [19,24]. Therefore, the amount of Al should be strictly controlled, creating the intractable “high Al-low toughness” bottleneck [23].

To break through the bottleneck, researchers have explored alternative microalloying elements. Ti and B elements precipitate with N and immobilize free N atoms in the weld metal [8,18]. The N content was about 200–500 ppm in the weld of semi-automatic welding, leading to the formation of coarse TiN and BN particles in the high-temperature molten pool, degrading the toughness [6]. The Zr element could refine TiN inclusions because the high-temperature ZrO_2_ particles could serve as nucleation sites of TiN inclusions [25], but the total N content does not decrease. The Cu element restrains the formation of granular bainite but promotes the formation of martensite, worsening the ductility and toughness [26]. Nb marginally improves the heat-affected zone cold cracking resistance even under low heat input parameters [27]. Mn is beneficial to the overall mechanical properties of the weld metal up to a certain limit, beyond which it promotes segregation and deteriorates the mechanical properties [9,28]. Unfortunately, all these elements either have a limited nitrogen-fixing capacity or introduce more detrimental side effects than Al. Notably, all existing approaches focus on immobilizing nitrogen through high-temperature precipitation, while the more fundamental strategy of reducing total nitrogen absorption during the welding process remains largely unexplored.

To date, no studies have systematically investigated alloying elements that can directly reduce nitrogen solubility in the semi-open molten pool of self-shielded welding. Existing research on Ni in pipeline welds has been exclusively limited to its microstructure-strengthening effects in gas-shielded welding processes, completely overlooking its potential role in regulating nitrogen behavior in the unique high-nitrogen metallurgical environment of self-shielded welding. This gap has prevented the development of a fundamental understanding of Ni’s full potential and hindered the design of high-toughness self-shielded flux-cored wires.

To address this gap, we designed eleven self-shielded flux-cored wires with Ni contents ranging from 1.42 wt.% to 4.02 wt.%. This work systematically investigates the effects of Ni content on weld continuous cooling transformation behavior, full-region microstructure evolution (solidification zone and all inter-pass heat-affected zones), nitrogen solubility and speciation, and comprehensive mechanical properties. The objective is to reveal the dual mechanism of Ni in simultaneously refining microstructure and reducing molten pool nitrogen solubility, providing data and theoretical support for optimizing the mechanical properties of multi-pass welds in low-alloy steels.

## 2. Materials and Methods

### 2.1. Materials Preparations

The composition of the commercial X80 pipeline steel is displayed in Table 1, depending on the ISO 3183:2019 [29] and GB/T 9711-2023 [30] PSL2 grade. Cr, Cu, and Ni are intentionally added to enhance corrosion resistance, while Ni and Mo are further incorporated to improve low-temperature toughness. In contrast to these major alloying elements, microalloying additions of Nb, V, and Ti are designed to precisely regulate austenite evolution during hot rolling and subsequent decomposition, thereby optimizing the microstructure and comprehensive mechanical properties, including fatigue resistance, stress corrosion cracking resistance, fracture toughness, and weldability.

The commercial X80 steel was manufactured by the following processes:

Smelting Process: First, the liquid iron produced via blast furnace smelting was charged into a 200-ton basic oxygen furnace (BOF) for primary steelmaking. Subsequently, the molten steel was refined in a ladle furnace (LF) for deep desulfurization, precise alloy composition adjustment, and liquid steel homogenization. Finally, deep degassing was performed in a Ruhrstahl–Heraeus (RH) vacuum degasser to effectively remove hydrogen and nitrogen, preventing defects such as blowholes and cracks while further reducing the steel carbon content.

Continuous Casting: Following continuous casting, slabs with a thickness of approximately 250 mm were produced. The casting speed was maintained at ~1 m/min, and the slabs were flame-cut into individual pieces.

Homogenization Treatment: The above billets underwent homogenization treatment at 1200 °C for 2 h.

**Figure 1:**
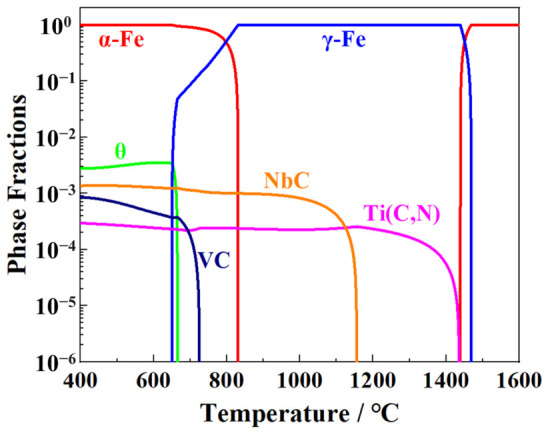
Property diagram of X80 steel calculated by Thermo-Calc software under the 2024a version.

TMCP Treatment: After homogenization, the slabs were hot-rolled into 18.4 mm-thick plates with a total rolling reduction of ~92.6%. To achieve complete dynamic recrystallization with minimal thickness reduction, the roughing start temperature was controlled at ~1100 °C, resulting in an intermediate slab thickness of ~95 mm. As shown in Figure 1, TiN starts to precipitate at ~1444 °C, forming coarse particles that do not affect dynamic recrystallization. In contrast, although NbC begins to precipitate at ~1156 °C, its inhibition effect on dynamic recrystallization in X80 steel only occurs above 950 °C. Accordingly, the finishing rolling start and finish temperatures were set at ~930 °C and ~830 °C, respectively. The roughing reduction was ~62%, and the non-recrystallized region rolling reduction reached ~80.6%. After hot rolling, the steel was water-cooled and coiled at 420 °C to promote the formation of a mixed microstructure consisting of acicular ferrite, bainite, quasi-polygonal ferrite, and martensite/austenite (M/A) constituents.

### 2.2. Welding Process

**Figure 2:**
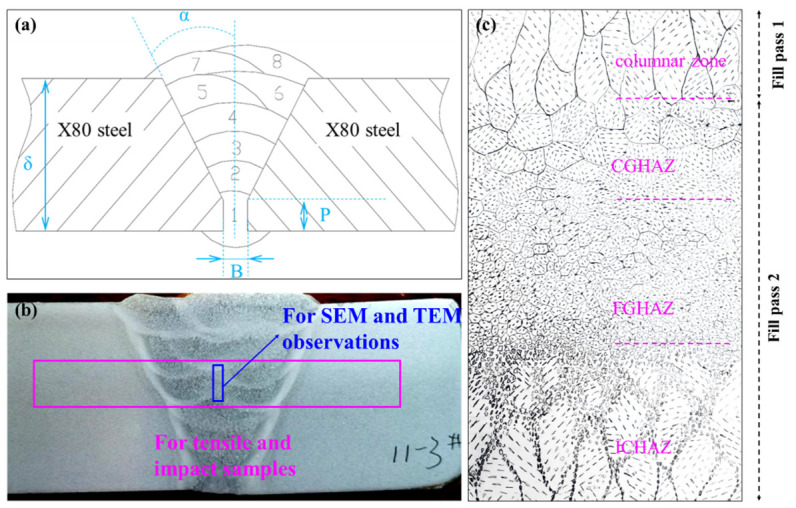
(**a**) Schematic diagram of girth welds of X80 steel welded by self-shielded flux-cored wire. (δ: 18.4 mm, P: 1 mm, α: 22°, and B: 3 mm); (**b**) the macrostructure of the weld joint under the welding wire K (11-3# is wire K); (**c**) Schematic diagram of the microstructural evolutions of different zones in the weld (CGHAZ: coarse grain heat-affected zone, FGHAZ: fine grain heat-affected zone, and ICHAZ: intercritical heat-affected zone).

Semi-automatic welding experiments were conducted on X80 steel plates using self-shielded flux-cored wires with systematically varied Ni contents under simulated vertical-down welding conditions. The root pass was deposited using a commercial BOEHLER SG3-P (voestalpine Böhler Welding Group GmbH headquartered in Düsseldorf, Suzhou, China) Φ1.2 mm CO_2_-shielded solid wire. The welding parameters for the fill and cap passes are summarized in Table 2, and the weld groove configuration and bead geometry are illustrated in Figure 2. The 11 wires with Ni content 1.42–4.02 wt.% systematically investigate the optimal Ni content, addressing the well-documented toughness fluctuation issue of standard commercial wires (Table 3).

For the root pass, the BOEHLER SG3-P Φ1.2 mm CO_2_-shielded solid wire with parameters was used: voltage 20–22 V, current 180–200 A, welding speed 15–18 cm/min, and CO_2_ flow rate 15–20 L/min. For fill and cap passes, the welding speed was 12–15 cm/min, corresponding to a controlled heat input of 1.0–1.2 kJ/mm, which is consistent with field engineering practices.

The 18.4 mm thick X80 steel plate was welded with 1 root pass, 6 fill passes, and 1 cap pass (8 passes in total). The inter-pass temperature was strictly maintained at 80–150 °C throughout the welding sequence. The macrostructure of the weld joint under the wire K was displayed in Figure 2b. Since wire K with the highest Ni content (4.02 wt.%) exhibited a favorable macroscopic weld morphology without obvious welding defects, and Ni is an austenite-stabilizing element that generally improves weldability by reducing cold cracking susceptibility rather than deteriorating it, it is inferred that welding wires with lower Ni content should also exhibit satisfactory weldability under the same welding parameters. Therefore, the macroscopic weld morphologies of lower Ni wires are not presented for brevity.

### 2.3. Continuous Cooling Transformation (CCT) Tests of the Weld

To investigate the phase transformation behaviors during weld solidification and cooling, two welding wires (F and J) with Ni contents of 2.56 wt.% and 3.73 wt.% in Table 2, respectively, were selected for the girth welding of X80 pipeline steel plates. Figure 2 shows a schematic diagram of girth welds of X80 steel welded by self-shielded flux-cored wire. The chemical composition of the weld metal, presented in Table 4, shows that the Ni content was significantly diluted compared to that in the corresponding flux-cored wires. This dilution is primarily attributed to the partial dissolution of the X80 base metal into the molten pool during welding. Subsequently, cylindrical thermal expansion specimens measuring Φ3 mm × 10 mm were extracted from the topmost cap pass layer for continuous cooling transformation (CCT) testing.

The continuous cooling transformation behavior of the weld was analyzed using the Formastor-F II (Fuji Electronic Industrial Co., Ltd., Tsurugashima, Saitama Prefecture, Japan) type thermal expansion instrument. The main process parameters were as follows: Heating to 1000 °C and holding for 30 s, and then continuous cooling to room temperature at the cooling rates of 0.06, 0.14, 0.28, 0.87, 1.74, 4.35, 8.7, 17.4, 43.5 °C/s for wire F and 0.06, 0.28, 0.92, 1.80, 4.6, 9.2, 18.4, 46.0 °C/s for wire J. The expansion curves obtained from the thermal expansion experiment were recorded.

### 2.4. Microstructure Characterizations

To reveal the microstructure, the specimens were prepared by grinding, polishing, and etching with 4 vol.% nital and were subsequently examined by Olympus GX51 (Olympus, Tokyo, Japan) optical microscopy (OM) and FEI Quanta 650 (Hillsboro, OR, USA) field emission scanning electron microscopy (SEM), respectively.

Sample preparation for transmission electron microscopy (TEM) analysis involved cutting 0.3 mm thick slices from the weld metals across different Ni levels of self-shielded flux-cored wires, followed by mechanical grinding to 50 μm and punching of 3 mm disks. Prior to examination, these disks underwent twin-jet electrolytic polishing at −20 °C and 30 mA in a 6 vol.% perchloric acid ethanol solution. A JEM-2100 TEM (JEOL Ltd., Tokyo, Japan) was utilized to observe the microstructure. The SEM and TEM samples were prepared from the blue rectangle area in Figure 2b.

### 2.5. N Content Tests in the Weld

Thermo-Calc software under the 2024a version, utilizing the TCFE 13 thermodynamics database, was employed to evaluate how the Ni element influences N element solubility within the molten pool. It is noted that the estimated N content by Thermo-Calc software is in an equilibrium state. The actual N content may differ from the calculated result; however, the relative relationship of the effect of Ni on the N element would not change.

The N content in the actual weld was tested by the oxygen–nitrogen analyzer.

### 2.6. Mechanical Property Tests

Preparation and testing of standard round tensile specimens were carried out along the rolling direction. These specimens, with dimensions of 50 mm gauge length, 10 mm diameter, and 110 mm total length, were subjected to uniaxial tension at 1 mm/min on a WE-300 machine at room temperature. The testing standard is GB/T 228.1-2021 [31], and the mean value of tensile properties depends on three samples.

Charpy V-notch impact toughness at −20 °C was assessed using a JBN-300B tester (Jinan Hengda Huifeng Test Instrument Co., Ltd., Jinan, China) on Charpy V-notch specimens (10 mm × 10 mm × 55 mm) machined along the rolling direction. The testing standard is GB/T 229-2020 [32], and the presented value represents the average of six parallel measurements. The tensile and impact samples were prepared from the pink rectangle area in Figure 2b.

Based on the testing standard of GB/T 4340.1-2024 [33], the Vickers hardness was estimated by the VH-5 Vickers hardness tester (Shanghai Everone Precision Instruments Co., Ltd., Shanghai, China). Five points were tested for average value. The specific parameters are as follows: the load is 5 kg and the load application time is 15 s.

## 3. Results and Discussions

### 3.1. Effect of Ni Element on the CCT Behaviors of the Weld

**Figure 3:**
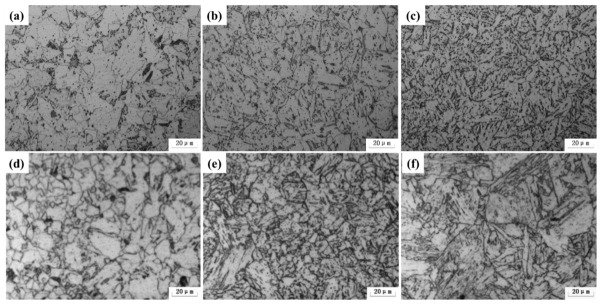
OM images showing the microstructural evolutions of the weld under the different cooling rate: Wire F with the Ni content of 2.56 wt.% under the cooling rates of (**a**) 0.28, (**b**) 4.6 and (**c**) 46 °C/s; Wire J with the Ni content of 3.73 wt.% under the cooling rates of (**d**) 0.14, (**e**) 4.35 and (**f**) 43.5 °C/s.

Figure 3a–c present the continuous cooling transformation (CCT) microstructures of the weld metal with 1.74 wt.% Ni (diluted from Wire F). With an increasing cooling rate, the weld microstructure showed progressive refinement of granular bainite and a simultaneous reduction in the size and number density of martensite/austenite (M/A) constituents. Specifically, at a cooling rate of 0.28 °C/s, the microstructure consisted of polygonal ferrite and pearlite with minor granular bainite. At 4.60 °C/s, it transformed to quasi-polygonal ferrite, granular bainite, and coarse M/A constituents, and finally became predominantly granular bainite at 45.60 °C/s.

Figure 3d–f present the CCT microstructures of the weld metal with 2.47 wt.% Ni (diluted from Wire J). These results revealed a complete microstructural transition from a mixed granular bainite–polygonal ferrite structure to fully lath bainite with an increasing cooling rate. Specifically, the initial near-equal mixture of the two phases at 0.14 °C/s evolved to incorporate lath bainite at 4.35 °C/s. The volume fraction of lath bainite increased at the expense of granular bainite, reaching complete conversion at 43.5 °C/s. Compared with the low-Ni weld, the high-Ni weld microstructure was significantly refined. Notably, large, irregularly shaped M/A constituents were almost entirely eliminated, while the remaining M/A constituents were refined and uniformly dispersed.

**Figure 4:**
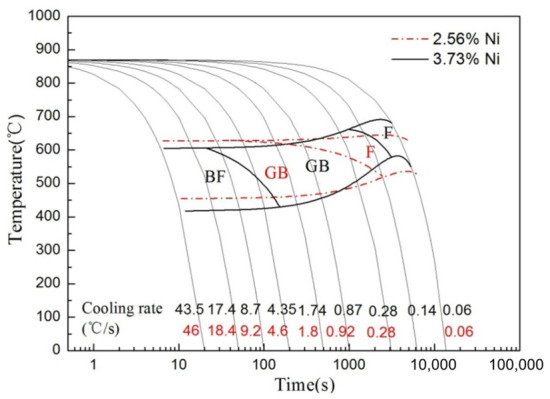
Comparisons of CCT curves of the weld under the wire F and wire J.

The CCT curves of the two welds by wire F and wire J are shown in Figure 4. The CCT curves of the weld were notably shifted by variations in Ni content. At a lower cooling rate, the increase in Ni content extended the ferrite phase transformation zone within the two factors of temperature and time. However, under higher cooling rate conditions, elevated Ni levels acted to lower the bainite starting and finishing transformation temperatures, which is the primary reason for the significantly refined microstructure of the weld based on the high-Ni welding wires.

Furthermore, the 1.74 wt.% Ni weld exhibited only two phase transformation regions, whereas the 2.47 wt.% Ni weld showed an additional lath bainite transformation region within the same cooling rate range. Comparative analysis of the CCT curves revealed that increasing Ni content shifted all phase transformation zones to the right along the time axis, significantly contracting the ferrite transformation region and narrowing the transition interval between the ferrite and granular bainite transformation zones. The most striking difference is that high Ni promoted lath bainite formation, while low Ni favored granular bainite formation.

Consequently, higher Ni levels in the weld depressed the bainite transformation temperature, resulting in a significant microstructure refinement effect. The Ni element enhanced the austenite hardenability, promoted the formation of lamellar bainite, reduced the M/A quantity and size, and optimized their morphology and distributions.

Ni is an element that increases the hardenability of high-strength low-alloyed steel. For the weld with the Ni content of 1.74 wt.% (Wire F), the microstructure contains polygonal ferrite and pearlite at a low cooling rate. However, no pearlite is found, and the polygonal ferrite size decreased in the weld with the Ni content of 2.47 wt.% (Wire J). Furthermore, at high cooling rates, the weld with the Ni content of 1.74 wt.% (Wire F) has low hardenability, so its microstructure is only granular bainite rather than lath bainite. By contrast, the lath bainite is observed in the weld with the Ni content of 2.47 wt.% (Wire J).

It is well established that Ni is an austenite-stabilizing element that expands the austenite single-phase region, thereby lowering the austenite decomposition temperature. As shown in Figure 4, the transformation temperature ranges for both lath bainite and granular bainite decrease with increasing Ni content. In the low-alloy chemical system of X80 steel, lath bainite typically forms at lower temperatures than granular bainite. This confirms that Ni stabilizes austenite and facilitates lath bainite transformation kinetics.

Ni not only reduces carbon activity and retards carbon diffusion but also promotes bainite formation. This promotion effect is most pronounced at the relatively high temperatures characteristic of granular bainite formation, where carbon atoms retain substantial diffusivity. During ferrite nucleation and growth, carbon can diffuse completely over long distances from the ferrite interior to the surrounding austenite and accumulate. In contrast, lath bainite forms at lower temperatures where carbon diffusion is severely inhibited, restricting carbon migration to short distances within ferrite laths.

Furthermore, granular bainite formation has a low thermodynamic driving force. Ferrite nucleates and grows irregularly and discretely at preferential sites such as austenite grain boundaries via a diffusion-controlled mechanism, gradually forming a polygonal ferrite matrix. In contrast, the lath bainite formation proceeds under a high thermodynamic driving force: ferrite nucleates in clusters along specific austenite crystallographic planes via a shear-dominated mechanism, resulting in lamellae containing high-density dislocations.

In granular bainite, the carbon-rich austenite regions retain some of their characteristics at room temperature due to their high carbon content and increased stability, and some transform into martensite. Eventually, isolated, coarse, granular, or “island-like” M/A constituents are randomly formed on the ferrite matrix. In lath bainite, the carbon that is inhibited from diffusing is concentrated between parallel ferrite plates, stabilizing the remaining austenite into continuous, slender, film-like M/A constituents that encapsulate each ferrite plate.

### 3.2. Effect of Ni Element on the Microstructural Evolutions of the Weld

To characterize the solidification microstructure evolution of weld metals produced using self-shielded flux-cored wires with systematically varied Ni contents, microstructural characterization was performed on the cap pass region of all welds.

**Figure 5:**
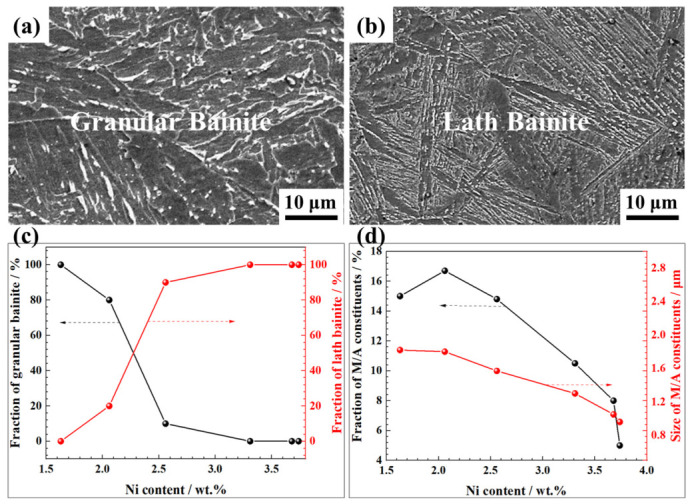
Microstructure of the weld metal solidification zone under varying Ni contents: SEM micrographs under the Ni content of (**a**) 1.63 wt.% and (**b**) 3.73 wt.%, respectively; (**c**) fractions of granular bainite and lath bainite with varying Ni content; (**d**) fractions and mean size of M/A constituents with varying Ni content.

As shown in Figure 5, the weld microstructure evolved progressively with the increasing Ni content. At 1.63 wt.% Ni, the weld metal consisted entirely of coarse granular bainite (Figure 5), whose M/A constituents exhibited a volume fraction of ~15 vol.% and an average size of ~1.80 μm (Figure 5d). When increasing the Ni content to 2.06 wt.%, lath bainite emerged, reducing the granular bainite fraction to ~80 vol.% (Figure S1a). At Ni contents above 2.56 wt.%, lath bainite became the dominant phase (Figure S1b–d). Concomitantly, the M/A constituents within the residual granular bainite decreased in both volume fraction and average size, refining from ~1.80 μm at 1.63 wt.% Ni to approximately 0.80 μm at higher Ni levels (Figure 5c,d).

**Figure 6:**
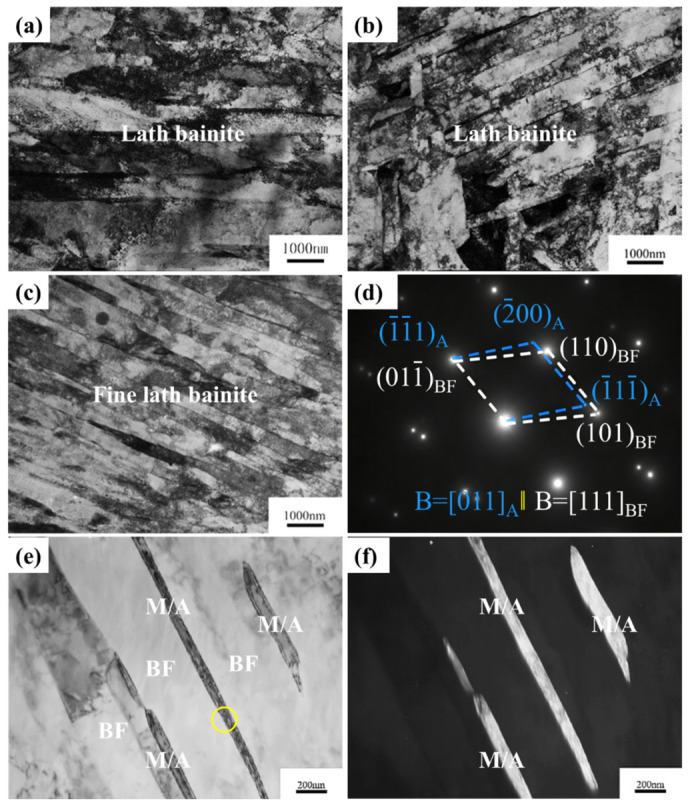
TEM micrographs of the weld metal solidification zone under varying Ni contents: (**a**) 2.06 wt.%, (**b**) 3.30 wt.%, and (**c**–**f**) 3.73 wt.%, respectively; (**a**–**c**) bright field images; (**d**) selected area electron diffraction pattern in the yellow circle in (**e**); (**e**) local magnification of bright field image (**c**); (**f**) dark field image of (**e**).

The bainite lath width in the weld metal was 1.20, 0.85, and 0.53 μm at Ni contents of 2.06, 3.30, and 3.73 wt.%, respectively, indicating that Ni significantly refined the lath bainite microstructure (Figure 6a–c). Collectively, these observations demonstrate that Ni significantly enhances the hardenability of the weld metal, leading to substantial microstructure refinement. Furthermore, transmission electron microscopy (TEM) analysis of bainite lath interfaces in the weld metal identified thin residual austenite films (Figure 6d–f), indicating that Ni stabilizes the austenite within the M/A constituents. In Figure 6d, it was found that the residual austenite and bainite lath keep the Kurdjumov–Sachs orientation relationship.

In multi-pass girth welds, repeated thermal cycling between successive weld passes induces spatial variations in thermal history across different regions, resulting in the formation of a series of distinct microstructural sub-zones within the inter-pass heat-affected zone (HAZ). These are characterized as the coarse-grain HAZ (CGHAZ), fine-grain HAZ (FGHAZ), intercritical HAZ (ICHAZ), and subcritical HAZ (SCHAZ). Herein, the HAZs in the weld differ from those in the matrix. A schematic diagram of the microstructural evolutions of different zones in the weld is displayed in Figure 3c.

**Figure 7:**
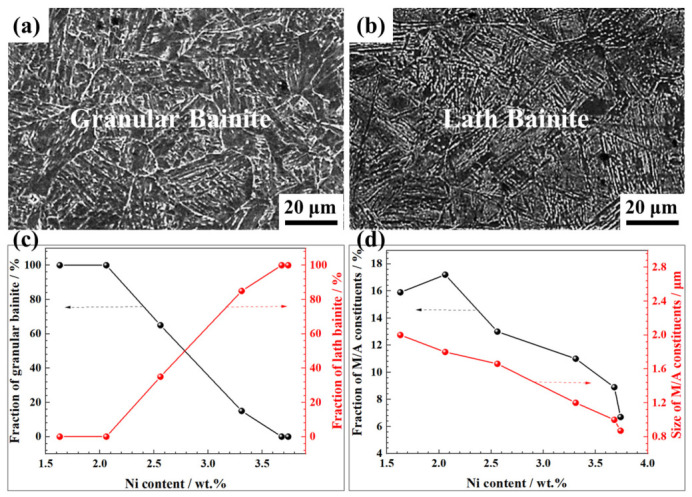
Microstructure of the CGHAZ in the weld under different Ni contents: SEM micrographs under the Ni content of (**a**) 1.63 wt.% and (**b**) 3.73 wt.%, respectively; (**c**) fractions of granular bainite and lath bainite with varying Ni content; (**d**) fractions and mean size of M/A constituents with varying Ni content.

The CGHAZ is characterized by prior austenite grains exceeding 50 μm (Figure 7). Within this zone, the microstructure progressively shifted from granular bainite towards lath bainite as the wire Ni content increased (Figure 7 and Figure S2). Specifically, a fully granular bainite structure persisted up to the Ni content of ~2.06 wt.% (Figure S2a). A mixed microstructure of granular bainite and lath bainite appeared at the Ni content between 2.56 and 3.30 wt.% (Figure S2b,c), transitioning to an almost completely lath bainite structure at the Ni content of 3.68 wt.% and above (Figure 7b and Figure S2d). Statistical analysis (Figure 7c,d) revealed contrasting characteristics of M/A constituents at different Ni levels. At wire Ni contents below 2.0 wt.%, the coarse-grained heat-affected zone (CGHAZ) contained >15 vol.% elongated M/A constituents with an average size exceeding 2.00 μm. In contrast, at wire Ni contents above 3.41 wt.%, the CGHAZ microstructure consisted of lath bainite with finely dispersed spherical M/A constituents distributed between the laths. Correspondingly, the M/A volume fraction was <10 vol.% and the average particle size decreased to <1.00 μm. At intermediate Ni contents, both the volume fraction and average size of M/A constituents decreased gradually.

**Figure 8:**
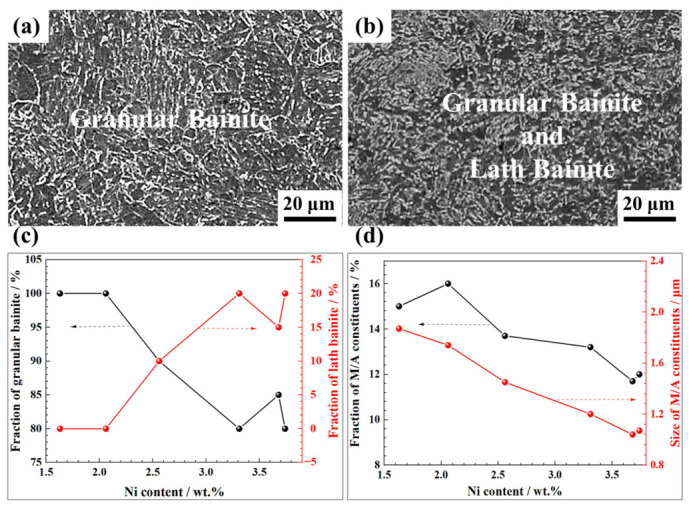
Microstructure of the FGHAZ in the weld under different Ni contents: SEM micrographs under the Ni content of (**a**) 1.63 wt.% and (**b**) 3.73 wt.%, respectively; (**c**) fractions of granular bainite and lath bainite with varying Ni content; (**d**) fractions and mean size of M/A constituents with varying Ni content.

As shown in Figure 8, the formation of the fine-grained heat-affected zone (FGHAZ) in the inter-pass reheating zone of the weld is attributed to the lower peak temperature of the weld thermal cycle compared to that in the coarse-grained heat-affected zone (CGHAZ), which reduces the thermodynamic driving force for austenite grain growth. During cooling, fine austenite grains exhibit lower thermal stability and preferentially decompose into ferrite at higher temperatures, resulting in the formation of polygonal ferrite in the FGHAZ.

At wire Ni contents below 2.06 wt.%, the FGHAZ consisted entirely of granular bainite (Figure 8a). As Ni content exceeded 2.06 wt.%, lath bainite began to form in the FGHAZ, and its volume fraction increased progressively with the increasing Ni content (Figure 8b and Figure S3a–d). Partial ferrite transformation still occurred due to the lower thermal stability of fine prior austenite grains. Consequently, even when the Ni content increased above 3.0 wt.%, the volume fraction of lath bainite in the FGHAZ remained limited to 15–20 vol.% (Figure 8c). Statistical analysis of the M/A constituents in the FGHAZ (Figure 8d) revealed a clear trend: both the volume fraction and average size of the M/A constituents decreased progressively with increasing wire Ni content.

**Figure 9:**
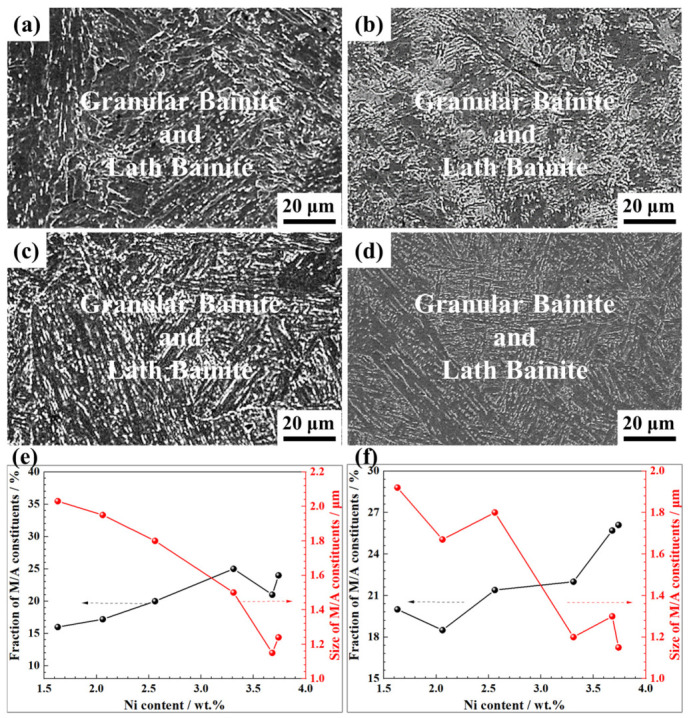
Microstructure of the ICHAZ and SCHAZ in the weld under different Ni contents: SEM micrographs of ICHAZ under the Ni content of (**a**) 1.63 wt.% and (**b**) 3.73 wt.%, respectively; SEM micrographs of SCHAZ under the Ni content of (**c**) 1.63 wt.% and (**d**) 3.73 wt.%, respectively; (**e**) fractions and mean size of M/A constituents with varying Ni content of the ICHAZ; (**f**) fractions and mean size of M/A constituents with varying Ni content of the SCHAZ.

The microstructure of the intercritical heat-affected zone (ICHAZ) in the inter-pass reheating region of the weld is inherently heterogeneous, characterized by extensive aggregation of M/A constituents (Figure 9a,c,e and Figure S4). With increasing Ni content, the inhomogeneous distribution of M/A constituents persisted, while their volume fraction increased and average size decreased (Figure 9e). At a wire Ni content of 1.56 wt.%, the average size of M/A constituents exceeded 2.0 μm. As Ni content increased to 3.73 wt.%, the average size of M/A constituents decreased to 1.2 μm. This phenomenon is attributed to the significant refinement of the weld microstructure at high Ni levels, which results in a higher number density of M/A constituents.

The microstructure of the subcritical heat-affected zone (SCHAZ) in the inter-pass reheating region exhibited a relatively high volume fraction of M/A constituents (Figure 9b,d,f and Figure S5). Statistical analysis revealed a positive correlation between increasing wire Ni content and the volume fraction of M/A constituents, accompanied by a concurrent decrease in their average size (Figure 9f).

The peak temperature of SCHAZ was below the A_1_ transformation temperature. Therefore, no austenitization occurs in SCHAZ. The coarse M/A islands within the original columnar grain structure appeared to coarsen and grow, whereas the smaller M/A islands dissolved or were engulfed by larger counterparts. The content of the M/A islands in SCHAZ typically ranged from 20 vol.% to 40 vol.%, exhibiting a size of approximately 2.5 μm or larger. Morphologically, these M/A islands were predominantly blocky, thick, or rod-like. Comparative micrographs of the SCHAZ microstructure and the original columnar grain structure are presented in Figure 9.

### 3.3. Effect of Ni Element on the Mechanical Properties of the Weld

**Figure 10:**
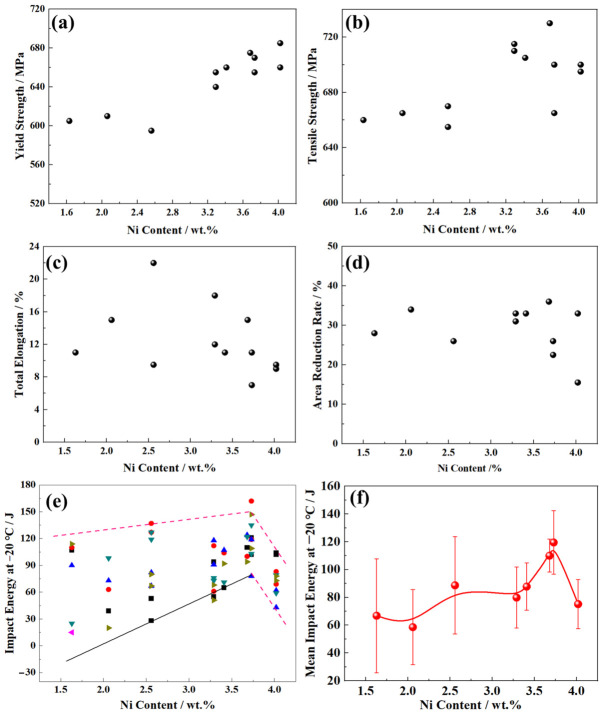
Mechanical properties of weld metals as a function of Ni content: (**a**) Yield strength; (**b**) tensile strength; (**c**) total elongation; (**d**) reduction in area. Each scatter point represents an individual test result (three parallel specimens per Ni content for tensile tests), and the solid line shows the mean value trend. (**e**) −20 °C Charpy V-notch impact energy. Each scatter point represents an individual impact test result (six parallel specimens per Ni content), directly illustrating the dispersion of impact toughness values. (**f**) Mean −20 °C Charpy impact energy. The solid line shows the average value of three parallel specimens, and the error bars represent the standard deviation of the measurements.

All mechanical property data are presented with individual test points to show the full distribution of results, alongside mean value curves to illustrate the overall trend with increasing Ni content. Error bars in Figure 10f represent the standard deviation of triplicate Charpy impact tests. An investigation into the effect of Ni content on weld metal mechanical properties involved the semi-automatic welding of X80 steel with 11 variant Ni-content self-shielded flux-cored wires, followed by comprehensive mechanical characterization (Figure 10).

The base X80 steel exhibits a yield strength of 695 MPa and a tensile strength of 784 MPa, corresponding to a yield ratio of 0.88. The total elongation and area reduction are about 22.2 and 37.6%. The impact energy at −20 °C is about 246 J. After welding, both the tensile properties and impact toughness decrease.

Ni content exhibited distinctly different effects on yield strength and tensile strength. For yield strength (Figure 10a), it remained stable at approximately 600 MPa until Ni content reached 2.56 wt.%, followed by a continuous increase to >660 MPa with further Ni additions. In contrast, Ni had a negligible effect on tensile strength below 2.56 wt.%, with values remaining stable around 660 MPa (Figure 10b). Above 2.56 wt.% Ni, tensile strength increased to ~730 MPa, but no significant further increase was observed with additional Ni.

For Ni contents below 3.30 wt.%, the elongation of the weld metal showed considerable fluctuations, while the reduction in area remained relatively constant at approximately 30% (Figure 10c,d). When Ni content exceeded 3.30 wt.%, both elongation and reduction in area decreased sharply. At 3.73 wt.% Ni, elongation reached a minimum of ~8%, and the reduction in area dropped to nearly 20% (Figure 10c,d).

The −20 °C impact energy of the weld metal peaked at a Ni content of 3.73 wt.%, following an overall increase with higher Ni levels (Figure 10e). A further increase in Ni content above 3.73 wt.% led to a significant decrease in −20 °C impact energy. Concurrently, the fluctuation range of −20 °C impact energy tended to narrow with increasing Ni content. For Ni contents below 2.56 wt.%, the −20 °C impact energy fluctuated widely, with an average of ~88.7 J. The −20 °C impact energy at 3.73 wt.% Ni ranged from 78 to 162 J, with a minimum of 119.5 J, while the overall average displayed an increase-then-decrease trend with Ni content, varying between roughly 58.6 J and 119.5 J. This plot delineates the relationship between higher Ni contents and impact energy (Figure 10f), clarifying the non-linear trend and specific value ranges.

At Ni contents below 2.06 wt.%, the microstructure of all interpass reheating zones in the weld metal is dominated by coarse granular bainite, resulting in a relatively low weld strength (Figure 10a,b). This is primarily attributed to two factors. Firstly, the granular bainite is a coarse polygonal bainite ferrite matrix, which has a weak grain boundary strengthening effect. Secondly, the low Ni content imparts a limited solid solution strengthening effect on the bainite ferrite matrix. For Ni contents ranging from 2.06 wt.% to 2.56 wt.%, the weld contains some lath bainite, and the microstructure gradually refines by a relatively slow refinement rate. A concurrent, yet modest, increase was observed in both the yield and tensile strength of the weld metal (Figure 10a,b). This trend arises from the gradual enhancement of both grain boundary strengthening and solid solution strengthening, which jointly drive the progressive increase in yield strength and tensile strength. However, when the Ni content exceeds 2.56 wt.%, the volume fraction of granular bainite decreases in all inter-pass re-heating zones of the weld metal, while that of lath bainite increases. Notably, the microstructure of the inter-pass SCHAZ appears significantly refined, which in turn leads to a marked enhancement of grain boundary strengthening.

When the Ni content is below 2.56 wt.%, the microstructure in the weld is dominated by coarse granular bainite, accompanied by many coarse M/A constituents. The austenite/martensite interface exhibits strong lattice distortion, which induces significant stress concentration and thus promotes crack initiation at these interfaces, substantially reducing the low-temperature toughness. Additionally, the martensite content in M/A constituents varies significantly with composition or microstructural state. Therefore, a higher content of M/A constituents in the weld not only leads to low impact toughness but also large toughness fluctuations. This explains why the toughness fluctuation range reaches up to 120 J within this composition range (Figure 10e).

As the Ni content exceeds 2.56 wt.%, both the volume fraction and size of M/A constituents decrease with an increasing proportion of lath bainite, resulting in increased impact energy and a narrowed fluctuation range (Figure 10e). At a Ni content of 3.73 wt.%, the weld metal consists primarily of significantly refined lath bainite (except for the inter-pass ICHAZ and some FGHAZ), with a small number of fine and dispersed M/A constituents. The microstructural refinement enhances the grain boundary strengthening effect, and Ni provides a more solid solution strengthening effect, while the refinement of M/A constituents mitigates stress concentration. Consequently, the weld metal exhibits both high strength and superior impact toughness (Figure 10a,b,e).

The systematic correlation between M/A constituent characteristics (size, volume fraction, morphology) and impact toughness has been demonstrated by experimental observations. The observed refinement of M/A constituents from coarse blocky structures to fine dispersed particles with increasing Ni content explains the improved toughness, which is consistent with numerous published studies on pipeline steel welds. The role of free nitrogen in degrading toughness is strongly supported by quantitative nitrogen content measurements showing that the lowest free nitrogen content occurs at the optimal Ni content of 3.73 wt.%, which exhibits an excellent correlation with the peak impact energy and minimum toughness dispersion. While direct fracture surface analysis would provide definitive confirmation of crack initiation and propagation mechanisms, the systematic quantitative correlations observed between microstructural characteristics (bainite type, M/A constituent size and distribution), nitrogen speciation (total, precipitated, and free nitrogen), and mechanical properties collectively provide strong supporting evidence for the proposed toughness improvement mechanisms.

Besides the change in Ni content, other alloying elements of the welding wire also present compositional differences. Hence, it is essential to investigate the variation regularities and contribution degrees of other element fluctuations to the evolution of mechanical properties.

Al (0.80–1.18 wt.% in wires, <0.8 wt.% in welds): While Al varies most significantly in wires, base metal dilution reduces its variation in actual welds to below 1%. Al’s effect on nitrogen solubility is opposite to Ni’s (Al increases N solubility), yet a clear decrease in total and free N with increasing Ni is still observed. This confirms that Ni’s effect dominates over Al’s.

C (0.028–0.043 wt.% in wires, <0.03 wt.% in welds): Dilution reduces C variation to <3%, which is too small to cause the observed 50% increase in impact energy or complete microstructural transition from granular to lath bainite.

Mn, Si, Zr, P, S: All show <15% variation in wires and <10% in welds. Their individual effects on the observed mechanical property trends are well-documented to be negligible at these concentration ranges. Therefore, within the present wire design space where other alloying elements show limited variations (<15% in wires and <10% in welds), Ni is the dominant contributor to the observed mechanical property evolutions within the present design, rather than implying a fully isolated single-variable effect.

### 3.4. Mechanism of Ni Element in Inhibiting the Harmful Role of the N Element in the Weld

During welding, the high dissolved nitrogen content in the molten pool reacts with alloying elements to form numerous polygonal nitride inclusions, which significantly degrade weld toughness. Furthermore, the high solubility of nitrogen in liquid steel leads to weld defects such as porosity and shrinkage cavities, further deteriorating weld toughness. These two mechanisms constitute the primary detrimental effects of nitrogen in weld metals.

Thus, regulating the solubility of N in the molten pool is essential. Alloying elements can modify the activity coefficient of the N atom, thereby influencing its solubility. The magnitude of this influence varies among different alloying elements. Elements with a strong affinity with N reduce the activity coefficient of N, leading to an increase in its solubility. The solubility of N in the liquid steel adheres to the following equation:

(1) $$\left[N\right]\;=\;K_{N}\sqrt{P_{N_{2}}}$$

Here, K_N_ represents the equilibrium constant of N dissolution, and $P_{N_{2}}$ is the partial pressure of N_2_ in the liquid steel. When alloying elements are added, the solubility of N in the liquid steel is expressed by the following equation:

(2) $$\left[N\right]\;=\;f_{N}\cdot K_{N}\sqrt{P_{N_{2}}}$$

Here, $f_{N}$ is the activity coefficient of the N element.

When the activity of N decreases, $f_{N}$ < 1, and the solubility of N in the molten pool [N] increases. In the molten pool, due to the presence of many alloying elements, $f_{N}$ is jointly controlled by multiple alloying elements. According to the Siverts law, $f_{N}$ is expressed by the following formula:

(3) $$\log f_{N}=e_{N}^{C}\left[\%C\right]+e_{N}^{\mathrm{Si}}\left[\%\mathrm{Si}\right]+e_{N}^{\mathrm{Mn}}\left[\%\mathrm{Mn}\right]+e_{N}^{P}\left[\%P\right]+e_{N}^{S}\left[\%S\right]+e_{N}^{V}\left[\%V\right]+e_{N}^{\mathrm{Nb}}\left[\%\mathrm{Nb}\right]\;+e_{N}^{\mathrm{Ni}}\left[\%Ni\right]$$

Here, e denotes the interaction coefficient between N and the alloying elements. Ishii, F. et al. [34] derived the first-order and second-order interaction coefficients for N with common alloying elements. However, since the low-alloyed steel investigated in this study has a low-alloying element content, the second-order interaction between N and the alloying elements is neglected. Additionally, $f_{N}$ is temperature-dependent, and its variation with temperature can be described by the following equation [35]:

(4) $$f_{N,T}=\left(\frac{3280}{T}-0.75\right)f_{N,1873}$$

Here, f_N,1873_ refers to the activity coefficient of N at a temperature of 1873 K [35].

Based on the Pehlke–Elliott theory [36], the equilibrium constant for N dissolution can be expressed as follows:

(5) $$\mathrm{lg}K_{N}=-\frac{188.052}{T}-1.17$$

Combining Equations (2)–(5), the expression for the solubility of N in liquid steel at different temperatures is obtained as follows:

(6) $$\mathrm{lg}\left[\%N\right]=\frac{1}{2}\mathrm{lg}\left(\frac{P_{N_{2}}}{P0}\right)-\frac{188.052}{T}-1.17-\left(\frac{3280}{T}-0.75\right)\left\{\left(\begin{array}{c} \&0.13\left[\%C\right]+0.047\left[\%\mathrm{Si}\right]\\ \&-0.02\left[\%\mathrm{Mn}\right]+0.024\left[\%\mathrm{Ni}\right]\\ \&+0.045\left[\%P\right]-0.093\left[\%V\right]\\ \&-0.06\left[\%\mathrm{Nb}\right] \end{array}\right)\right\}$$

In Equation (3), a negative interaction coefficient (e) leads to an increase in the solubility of N in the molten pool, whereas a positive (e) results in a decrease in N solubility. Additionally, N solubility in the molten pool rises with increasing temperature. During welding, the molten pool temperature can exceed 2000 °C, at which nitrogen solubility in liquid steel is significantly elevated. Therefore, reducing nitrogen solubility through alloying is a critical control strategy. According to Equation (6), increasing the carbon content in the welding wire or flux core lowers nitrogen solubility in the molten pool. Furthermore, the reaction of carbon with oxygen to form CO and CO_2_ improves molten pool protection, reducing atmospheric nitrogen ingress. Elements such as Ni, Si, and P also decrease nitrogen solubility, whereas Mn and Nb increase it. However, excessive addition of non-metallic elements (C, Si, P) in flux-cored wires significantly deteriorates the impact toughness of weld metals. Consequently, reducing nitrogen solubility through this approach is not optimal.

**Figure 11:**
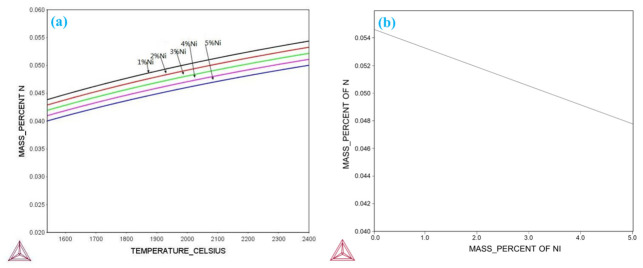
Thermo-Calc simulations in equilibrium state: (**a**) Effects of temperature and Ni content on the solid solubility of N in the molten pool and (**b**) effect of Ni content on the solid solubility of N in the molten pool at 2000 °C.

Notably, Ni not only improves the strength and toughness of the weld metal but also reduces N solubility. Investigating the influence of Ni on the N solubility is therefore of substantial significance. Thermo-Calc thermodynamic software was employed for a detailed analysis of the relationship between Ni content and N solubility in the molten pool. As shown in Figure 11a, N solubility decreases significantly with a reduction in molten pool temperature. Additionally, the content of the dissolved N element in the molten pool increases as the Ni content in the weld metal decreases (Figure 11b).

**Figure 12:**
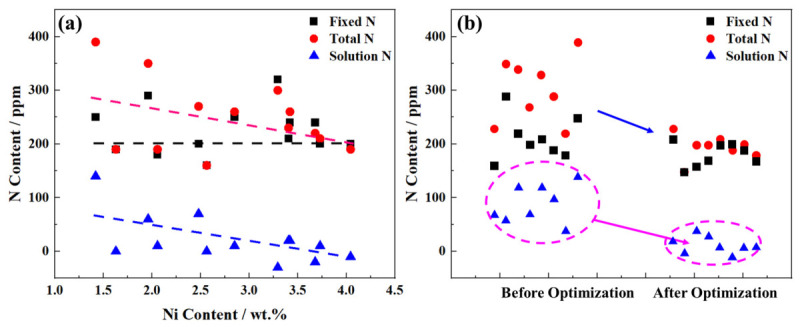
(**a**) Effect of Ni element on the content of different existence forms of N element in the weld (the dotted line represents the average N content in the weld), and (**b**) the variation trend of solid solution N content in the weld seam before and after optimization by adding 3.73 wt.% of the Ni element.

During welding, the molten pool temperature typically exceeds 2000 °C, corresponding to an N content of over 450 ppm. From thermodynamic analysis, increasing the Ni content from 1 wt.% to 5 wt.% reduces the N content by 40–50 ppm. To investigate the effect of Ni content in the actual flux-cored wire powder on the solid solubility of N in the weld, 11 flux-cored wire formulations with varying Ni contents were adopted. The Ni content in the deposited metal of these wires ranged from 1.6 wt.% to 4.1 wt.%. Thermodynamic calculations are only obtained under equilibrium conditions, and the experimentally measured total dissolved nitrogen content (<400 ppm) is lower than the Thermo-Calc predicted value (>450 ppm). The free nitrogen content in the weld was derived by subtracting the precipitated N (quantified via extraction chemical phase analysis) from the total N (measured with an O/N analyzer). This analytical procedure is summarized in Figure 12a.

The non-uniform distribution of nitrogen in weld metals originates from two primary mechanisms. First, the heterogeneous precipitation and distribution of nitrides during molten pool solidification. Second, the tendency of free nitrogen atoms to aggregate at micropores or segregate along dislocation lines to form nitrogen-enriched channels when the free nitrogen content exceeds a critical threshold. This heterogeneity causes significant fluctuations in measured total nitrogen content, particularly at elevated total nitrogen levels (Figure 12a).

While the total nitrogen content decreases with increasing Ni content, precipitated nitrogen content remains nearly constant at ~200 ppm. Notably, free nitrogen content in the weld metal decreases progressively with increasing Ni content, approaching zero when Ni content exceeds 3.5 wt.% (Figure 12a). These results confirm that increasing the Ni content in self-shielded flux-cored wires effectively reduces total nitrogen content, particularly free nitrogen content. This observation is consistent with Thermo-Calc thermodynamic calculations, which demonstrate that Ni lowers nitrogen solubility in the molten pool (Figure 11b).

The primary motivation was to explicitly illustrate how Ni content in self-shielded flux-cored wires affects the nitrogen content in X80 steel girth welds. To this end, a direct comparison was made between the N levels in welds fabricated with the baseline wire and those produced with Ni-optimized wires (Figure 12b). The total N content of the original welds ranged from 200 to 400 ppm, whereas after optimizing the Ni content in the welding wire, the total N content of the welds was reduced to below 200 ppm. For the original X80 self-shielded flux-cored wire girth welds, the free N content exhibited large fluctuations (30–140 ppm in Figure 12b). In contrast, the optimized X80 self-shielded flux-cored wire girth welds showed a marked reduction in free N content (below 50 ppm in Figure 12b), which effectively mitigates the risk of micropore or crack formation caused by free N segregation. This improvement plays a crucial role in mitigating the variability of impact toughness in X80 girth welds, and it also explains why the variability in weld metal impact toughness decreases gradually with increasing Ni content (Figure 10e).

When the Ni content in the welding wire is increased from 1.5 wt.% (present Ni content level) to about 3.7 wt.%, the alloy cost per ton of welding wire increases by 13–15% after adding a reasonable profit margin. Since welding consumables only account for 1.2–1.8% of the total construction cost of long-distance pipelines, the overall project cost only increases by 0.18–0.27%.

The 3.73 wt.% Ni in the welding wire corresponds to a diluted Ni content of 2.47 wt.% in the weld metal, with a carbon equivalent of 0.495%, which is comparable to that of the X80 base metal. A sharp drop in toughness due to large blocky retained austenite only occurs when Ni exceeds 3.8 wt.%, and this boundary has been explicitly avoided in this study. It is fully compatible with existing self-shielded flux-cored wire production lines without any equipment modification, only requiring optimization of the mixing uniformity control of the Ni element. The welding parameters are completely consistent with those of conventional welding wires, and construction personnel do not need retraining.

In summary, the significant improvement of impact toughness of girth welds induced by Ni stems from the synergistic effect of two mechanisms, refining the size and enhancing the distribution uniformity of M/A constituents, while simultaneously reducing the free N content in the weld.

## 4. Conclusions

In this work, eleven self-shielded flux-cored wires with varying Ni contents were designed to optimize the comprehensive mechanical property of X80-grade pipeline steel girth welds, yielding the following key conclusions:

(1) Ni lowers the phase transformation temperatures of both lath bainite and granular bainite, and significantly refines the lath bainite microstructure. Meanwhile, the formation of coarse M/A constituents is effectively inhibited.

(2) As the Ni content increases from 1.42 wt.% to 4.02 wt.%, the dominant microstructure of X80 pipeline steel girth welds evolves gradually from coarse granular bainite to fine lath bainite. Concurrently, the M/A constituents became finer and more uniformly dispersed, with their average size decreasing from ~2 μm to ~1 μm.

(3) Increasing the Ni content leads to a gradual increase in both yield strength (from 600 MPa to 660 MPa) and tensile strength (from 660 MPa to 730 MPa), while the −20 °C Charpy impact energy reaches a peak value of 119.5 ± 22.8 J at ~3.73 wt.% Ni. Additionally, Ni significantly reduces the dispersion of impact energy values.

(4) Ni improves the impact toughness of X80 pipeline steel girth weld metal primarily through two synergistic mechanisms. First, Ni promotes the formation of lath bainite and refines M/A constituents, thereby reducing microscale stress concentration. Second, Ni lowers the solubility of N in the molten pool, mitigating the formation of micropores or microcracks caused by free nitrogen segregation.

(5) The self-shielded flux-cored wire with 3.73 wt.% Ni achieves an optimal combination of high strength and excellent low-temperature impact toughness for the X80 pipeline steel girth welds. This provides a clear compositional guideline for the development of high-performance self-shielded flux-cored wires.

## Figures and Tables

**Table 1:** Chemical composition of the commercial X80 pipeline steel (wt.%).

C	Si	Mn	Cr	Ni	Mo	Cu	Nb	Ti	V	Al	P	S
0.038	0.15	1.76	0.22	0.17	0.22	0.15	0.082	0.017	0.026	0.027	0.01	0.0018

**Table 2:** Welding process parameters for fill and cap passes.

Preheating Temperature/°C	Inter-Pass Temperature/°C	Voltage/V	Current/A	Feeding Speed/cm·s^−1^	Polarity
≥100	80~150	18~20	200~230	4.0	DCEN

**Table 3:** Chemical compositions of self-shielded flux-cored wires with different Ni contents (wt.%).

No.	Al	C	Co	Mn	Ni	P	S	Si	Ti	Zr
A	0.86	0.029	0.25	1.48	1.42	0.0098	0.0021	0.0309	0.004	0.044
B	0.80	0.035	0.28	1.69	1.63	0.0084	0.0025	0.0627	0.004	0.039
C	0.95	0.028	0.27	1.50	1.96	0.0098	0.0024	0.0339	0.005	0.051
D	0.86	0.033	0.28	1.70	2.06	0.0084	0.0027	0.0600	0.004	0.041
E	0.98	0.033	0.26	1.45	2.48	0.0097	0.0026	0.0428	0.004	0.055
F	1.01	0.038	0.27	1.69	2.56	0.0082	0.0026	0.0653	0.004	0.042
G	1.08	0.040	0.29	1.77	3.30	0.0083	0.0030	0.0662	0.004	0.051
H	1.11	0.042	0.27	1.71	3.41	0.0086	0.0031	0.0647	0.005	0.053
I	1.14	0.043	0.27	1.67	3.68	0.0090	0.0033	0.0695	0.004	0.054
J	1.18	0.036	0.29	1.73	3.73	0.0079	0.0032	0.0655	0.004	0.056
K	1.06	0.033	0.28	1.66	4.02	0.0077	0.0033	0.0637	0.004	0.052

**Table 4:** Chemical compositions in the weld (wt.%).

No	Al	C	Co	Cr	Cu	Mn	Mo	Nb	Ni	Si	V	Zr
F	0.88	0.035	0.193	0.09	0.067	1.67	0.065	0.027	1.74	0.14	0.012	0.047
J	0.877	0.036	0.198	0.077	0.059	1.59	0.051	0.022	2.47	0.13	0.010	0.044

## Data Availability

The original contributions presented in this study are included in the article/Supplementary Material. Further inquiries can be directed to the corresponding author.

## Supplementary Materials

The following supporting information can be downloaded at https://www.mdpi.com/article/10.3390/ma19102162/s1. Figure S1: SEM images of the weld metal solidification zone under varying Ni contents: (a) 2.06 wt.%; (b) 2.56 wt.%; (c) 3.30 wt.%; (d) 3.68 wt.%; Figure S2: SEM images of the CGHAZ in the weld under different Ni contents: (a) 2.06 wt.%; (b) 2.56 wt.%; (c) 3.30 wt.%; (d) 3.68 wt.%; Figure S3: SEM images of the FGHAZ in the weld under different Ni contents: (a) 2.06 wt.%; (b) 2.56 wt.%; (c) 3.30 wt.%; (d) 3.68 wt.%; Figure S4: SEM images of the ICHAZ in the weld under different Ni contents: (a) 2.06 wt.%; (b) 2.56 wt.%; (c) 3.30 wt.%; (d) 3.68 wt.%; Figure S5: SEM images of the SCHAZ in the weld under different Ni contents: (a) 2.06 wt.%; (b) 2.56 wt.%; (c) 3.30 wt.%; (d) 3.68 wt.%.
