# Supplementary material for: Effect of Ni Element in Self-Shielded Flux-Cored Wires on the Microstructural and Mechanical Property Evolutions of X80 Pipeline Steel Girth Welds
Source: Materials (Basel). 2026 May 21;19(10):2162. doi: 10.3390/ma19102162 (PMC13209082; doi:10.3390/ma19102162)
Supplement: Supplementary file 1 [file materials-19-02162-s001.zip › materials-4288632-supplementary.pdf]

*Supplementary Materials*

# Effect of Ni Element in Self-Shielded Flux-Cored Wires on the Microstructural and Mechanical Property Evolutions of X80 Pipeline Steel Girth Welds

**Shujun Jia [1,*], Chengwu Cui [2], Chunliang Mao [1], Gang Liu [1] and Qingyou Liu [1]**

1 Engineering Structural Steel Research Institute, Central Iron and Steel Research Institute Company Limited, Beijing 100081, China;
2 China Petroleum Pipeline Research Institute Company Limited, Langfang 065000, China;
* Correspondence: jiajsj504@126.com

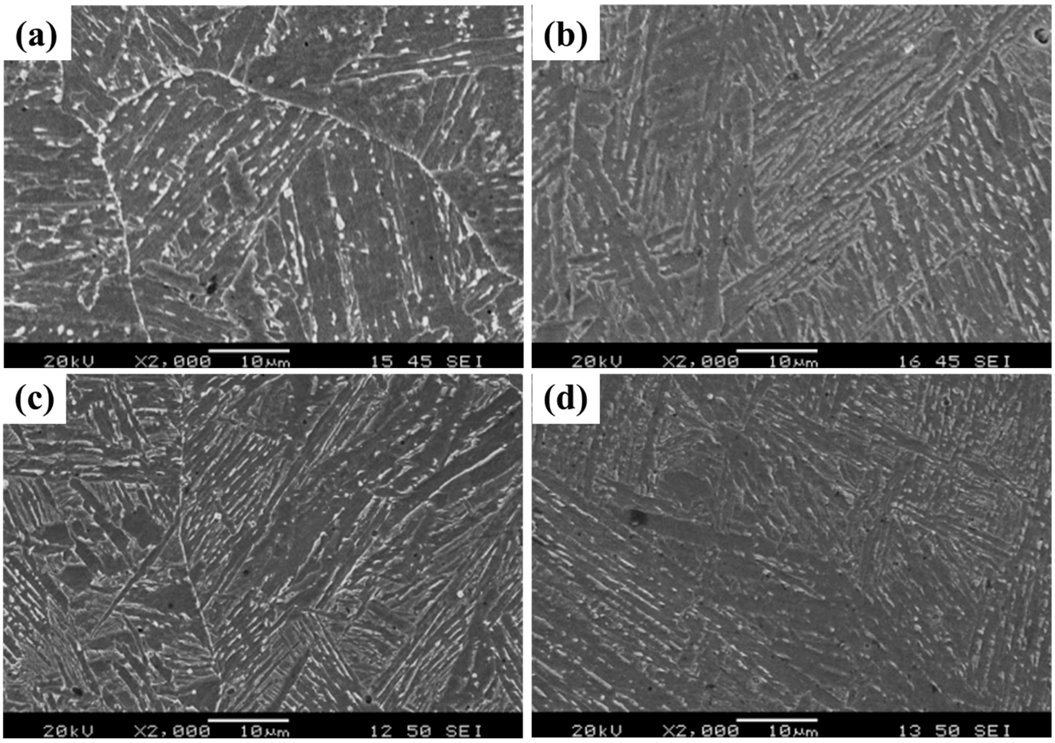


**Figure S1.** SEM images of the weld metal solidification zone under varying Ni contents: (a) 2.06 wt.%; (b) 2.56 wt.%; (c) 3.30 wt.%; (d) 3.68 wt.%.

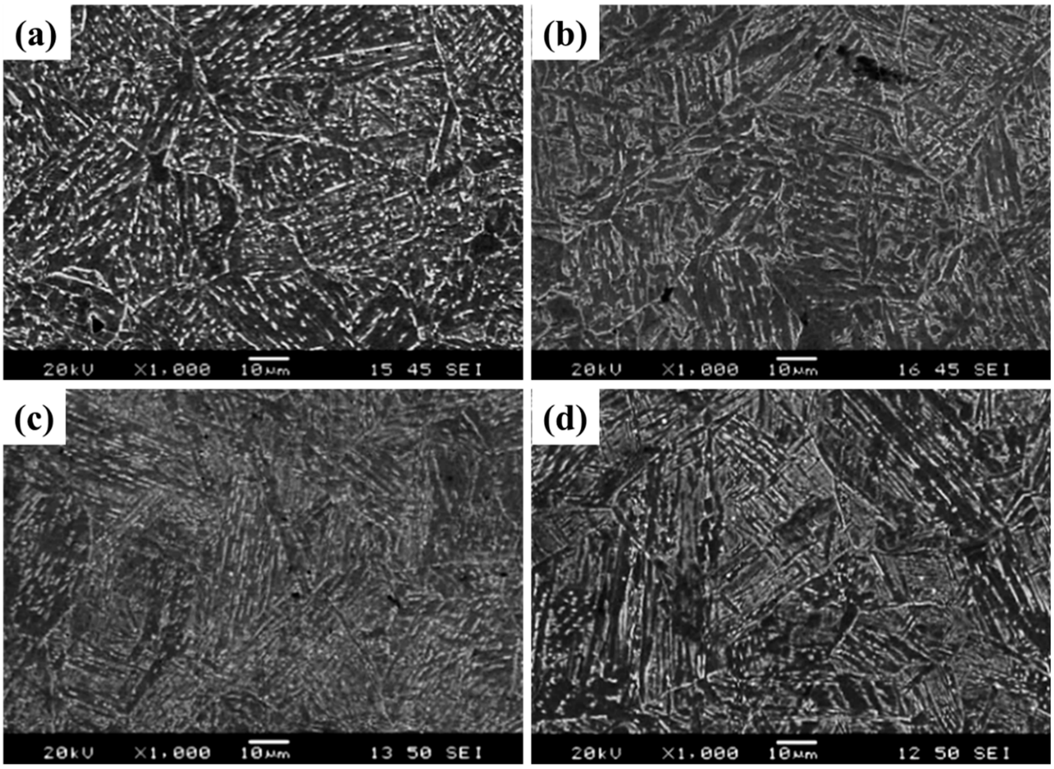


**Figure S2.** SEM images of the CGHAZ in the weld under different Ni contents: (a) 2.06 wt.%; (b) 2.56 wt.%; (c) 3.30 wt.%; (d) 3.68 wt.%.

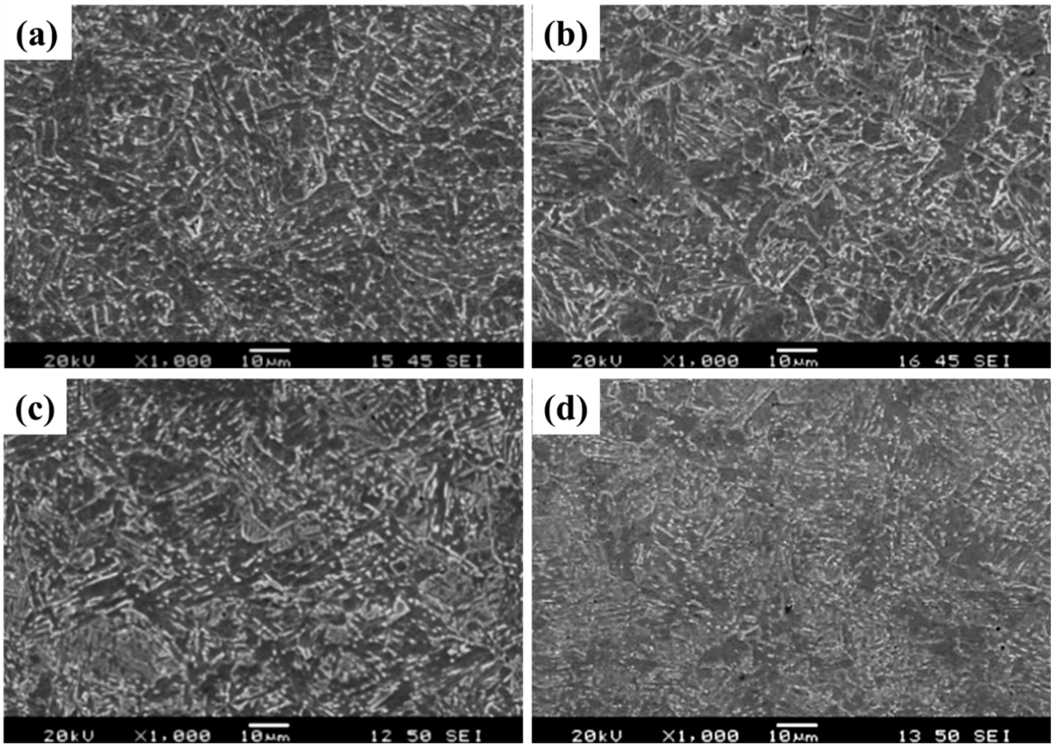


**Figure S3.** SEM images of the FGHAZ in the weld under different Ni contents: (a) 2.06 wt.%; (b) 2.56 wt.%; (c) 3.30 wt.%; (d) 3.68 wt.%.

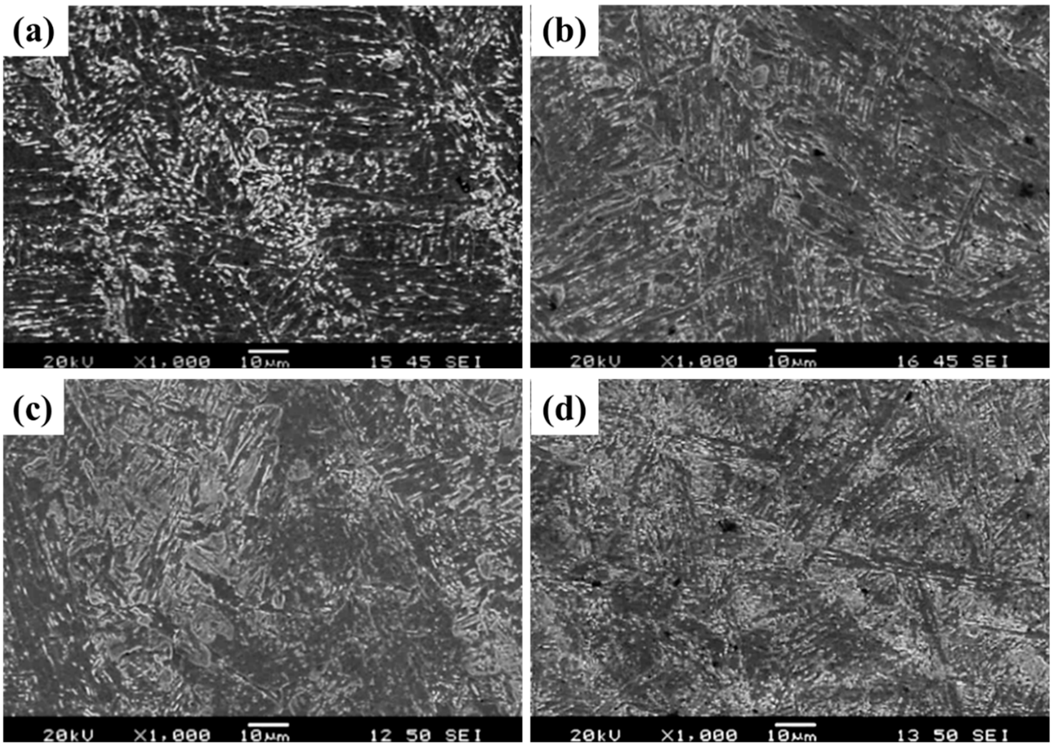


**Figure S4.** SEM images of the ICHAZ in the weld under different Ni contents: (a) 2.06 wt.%; (b) 2.56 wt.%; (c) 3.30 wt.%; (d) 3.68 wt.%.

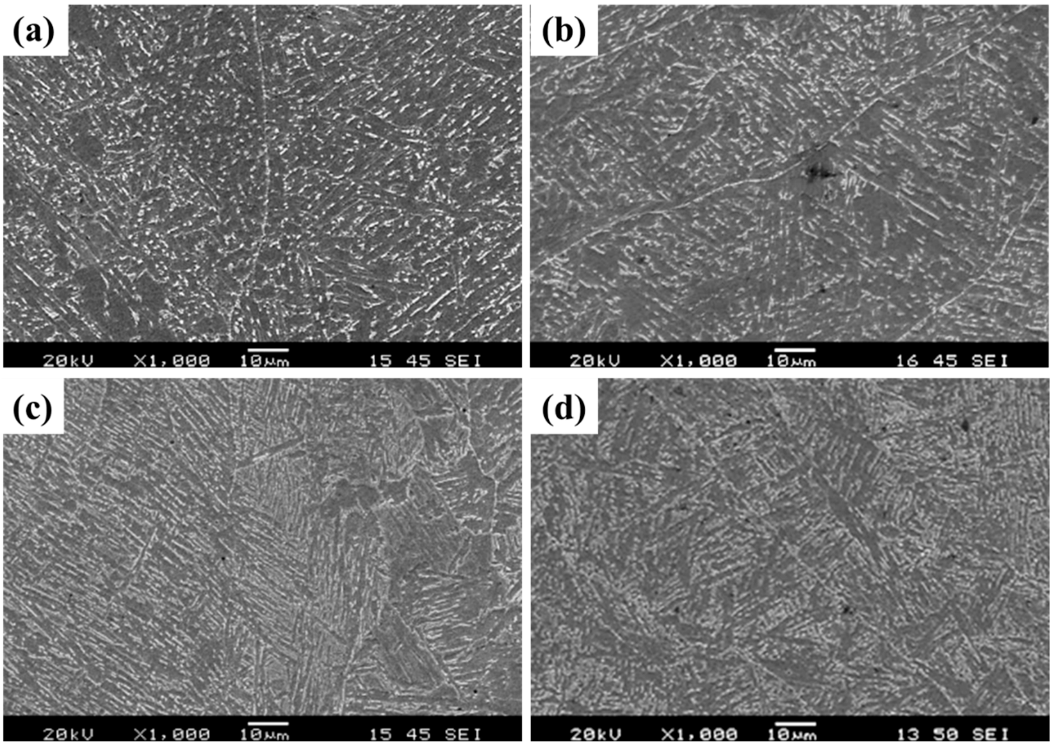


**Figure S5.** SEM images of the SCHAZ in the weld under different Ni contents: (a) 2.06 wt.%; (b) 2.56 wt.%; (c) 3.30 wt.%; (d) 3.68 wt.%.
